# Microstructures and mechanical characteristics of primary teeth affected by regional odontodysplasia

**DOI:** 10.1590/1678-7765-2025-0499

**Published:** 2026-01-12

**Authors:** Wenxiang Jiang, Minjian Shen, Jian Wang, Mengyao Bian, Haiyan Zheng, Zihuai Zhou, Ying Shi, Zhifang Wu

**Affiliations:** 1 Zhejiang University School of Medicine School of Stomatology Stomatology Hospital Hangzhou China Engineering Research Center for Oral Biomaterials and Devices of Zhejiang Province, Cancer Center of Zhejiang University, Key Laboratory of Oral Biomedical Research of Zhejiang Province, Zhejiang Provincial Clinical Research Center for Oral Diseases, Zhejiang University School of Medicine, School of Stomatology, Stomatology Hospital, Department of Pediatric Dentistry, Hangzhou, China.; 2 National Clinical Research Center for Child Health Zhejiang University School of Medicine Children's Hospital China National Clinical Research Center for Child Health, Zhejiang University School of Medicine, Children's Hospital, Hangzhou Department of Stomatology, China.; 3 Zhejiang University School of Medicine School of Stomatology Stomatology Hospital Hangzhou China Engineering Research Center for Oral Biomaterials and Devices of Zhejiang Province, Cancer Center of Zhejiang University, Key Laboratory of Oral Biomedical Research of Zhejiang Province, Zhejiang Provincial Clinical Research Center for Oral Diseases, Zhejiang University School of Medicine, School of Stomatology, Stomatology Hospital, Department of Prosthodontics, Hangzhou, China.

**Keywords:** Odontodysplasia, Primary teeth, X-Ray microtomography, Electron microscope tomography

## Abstract

Regional odontodysplasia (ROD) is a rare, localized disorder of tooth hard tissues that can affect primary and permanent dentitions. However, its definitive etiology remains unclear and only a few studies have reported the detailed microstructures and mechanical characteristics of the affected teeth.

**Objective:**

This study investigates the microstructural and mechanical characteristics of primary teeth affected by ROD.

**Methodology:**

A total of two ROD-affected primary teeth from two different cases underwent clinical examinations. In addition to control samples of caries-free retained primary teeth, the affected samples were examined using micro-computed tomography (micro-CT), X-ray diffraction (XRD), scanning electron microscopy (SEM), energy-dispersive X-ray spectroscopy (EDS), transmission electron microscopy (TEM), and nanoindentation analysis.

**Results:**

Clinical findings revealed yellowish discoloration, rough surfaces, and root resorption on the affected teeth. Radiographs indicated hypocalcified enamel, widened pulp chambers, and delayed development of permanent teeth. Micro-CT showed affected teeth with thinner and uneven enamel, disordered dentin, and reduced mineral density. XRD analysis found reduced crystallinity. SEM and TEM analyses revealed hypoplastic and loosely packed enamel crystals, whereas dentin exhibited disorganized collagen fibrils and poorly mineralized crystals. EDS analysis showed a reduced calcium/phosphorus ratio and an increased magnesium/calcium ratio in the affected enamel. Nanoindentation tests found reduced hardness and elastic modulus in ROD-affected enamel compared with control teeth.

**Conclusion:**

ROD-affected primary teeth display significant microstructural abnormalities and compromised mechanical properties, underscoring the need for early intervention and long-term monitoring to prevent complications.

## Introduction

Regional odontodysplasia (ROD) is a rare, localized developmental disorder of tooth hard tissues that can affect primary and permanent dentitions.^1,2^ ROD incidence is extremely low, with fewer than 200 cases documented worldwide. It generally manifests in a localized manner, affecting one or two jaw quadrants, predominantly in the anterior tooth region, and occasionally extending over the midline.^3,4^ Although ROD cases affecting multiple quadrants or a single tooth have been documented, they remain rare. ROD is more commonly observed in the maxilla compared to the mandible. Clinically, affected teeth usually exhibit a yellowish-brown discoloration, hypomineralization, and delayed eruption. Additionally, these teeth have abnormal morphology characterized by the presence of pits and grooves which predispose them to caries upon eruption.^5^ Radiographic analysis reveals that ROD-affected teeth present a poorly defined, thin, and hypomineralized layer of hard tissue, leading to the term “ghost teeth”.^6^ Typically, these teeth are distinguished by enlarged pulp chambers with calcifications and short roots with open apices.

Its definitive etiology remains unclear despite the numerous potential impact factors identified. Despite consensus that ROD is non-hereditary, some researchers proposed a possible genetic link.^7-9^ Various potential etiological factors have been considered, including local circulatory disorders, trace element imbalances, trauma, viral infections, somatic mutations, hyperthermia, radiation exposure, and medication use during pregnancy.^10-13^ However, none of these factors alone can comprehensively explain all cases, prompting some researchers to believe that multiple risk factors contribute to ROD.^3^ Typically, ROD diagnosis is straightforward and definitive due to its distinctive clinical and radiographic characteristics.^2^

Thoroughly understanding the structure and properties of the affected enamel and dentin is essential to optimize the outcomes of restorative and endodontic treatments, as these ROD-affected teeth are susceptible to caries and pulpal-periapical diseases. Although Hitch first described dysplasia 90 years ago, limited research has been conducted on the physical and morphological composition of ROD-affected teeth.^14-17^

Hence, this study investigated the microstructure and mechanical properties of ROD-affected primary teeth from two cases using high-resolution micro-computed tomography (micro-CT), scanning electron microscopy (SEM), energy-dispersive X-ray spectroscopy (EDS), X-ray diffraction (XRD), transmission electron microscopy (TEM), and nanoindentation analysis. Thus, it aims to provide an in-depth comprehension of the pathophysiological mechanisms contributing to the aberrant tooth structure observed in ROD and offer guidelines for clinical treatment.

## Methodology

This study included two patients diagnosed with ROD based on their clinical and radiographic features. A mandibular primary lateral incisor (tooth 82) from case 1 and a maxillary primary molar (tooth 54) from case 2, both ROD-affected, were extracted and used as subjects. While tooth 82 was selected for high-resolution micro-CT, SEM, EDS, XRD, and nanoindentation analysis, tooth 54 was used for TEM examination. A caries-free primary incisor (tooth 82) and primary molar (tooth 54), both identified as retained primary teeth, were extracted and used as control samples. All teeth were cleaned to remove debris, plaque, and soft tissue and then stored in 0.5% chloramine-T at 4 ℃ until testing. Ethical approval was obtained from the institutional review board of the Stomatology Hospital, Zhejiang University School of Medicine (Approval number: 2024026).

### High-resolution micro-CT analysis

A high-resolution micro-CT scanner (SkyScan 1272, Bruker, Kontich, Belgium) analyzed the affected and control primary incisors. The X-ray operated at 80 kV and 100 µA, with a 1-mm-thick aluminum filter placed in the beam path. Resolution was set at 9 µm pixel size, with a rotation step of 0.6 degrees. Three-dimensional (3D) images were reconstructed using NRecon software (SkyScanTM, Bruker, Belgium) and analyzed on CTAn software (SkyScanTM, Bruker, Belgium). Mean greyscale level of each tooth region (such as enamel and dentin, n=10) was calculated and subsequently transformed into mineral density values using the calibration equation established by standard hydroxyapatite phantoms.^18^

### XRD analysis

Crystallinity of the enamel mineral was evaluated by X-ray diffraction (XRD) using an X-ray diffractometer (XPERT-3, PANalytical, the Netherlands) operating at 40 kV and 40 mA. Diffraction patterns were collected throughout a 2θ range of 10° to 70° with a step size of 0.026°. MDI Jade 6 software (MDI, Livermore, CA, USA) analyzed the data for lattice parameters and crystallinity.

### SEM and EDS analysis

The enamel, dentin, and cementum samples obtained from the incisors were analyzed using SEM (GeminiSEM300, Zeiss, Jena, Germany). EDS analyses were performed on the enamel surface to determine the relative concentrations of calcium (Ca), phosphorus (P), and magnesium (Mg) using a Bruker EDS detector (X-Flash 6|30, Bruker, MA, USA) (n=15).

### TEM analysis

Enamel and dentin specimens obtained from the ROD-affected and control primary molars were fixed using Karnovsky’s fixative and postfixed with 1% osmium tetroxide. Each specimen was then rinsed three times with phosphate buffer saline, and subsequently dehydrated using an ascending series of ethanol (30%−100%) and acetone. Finally, the samples were embedded in epoxy resin overnight at room temperature. Ultra-thin sections (about 70−90 nm) were observed using a JEM 1230 TEM (JEOL, Tokyo, Japan) operating at 80 kV.

### Nanoindentation analysis

The ROD-affected and control primary incisors were sectioned using a low-speed diamond saw to obtain enamel surface samples for nanoindentation analysis. Nanoindentation tests were conducted using a Nano Indenter G200 (Agilent, Santa Clara, CA, USA) with a diamond Berkovich tip. Each sample had 15 indentation points arranged in a 3 × 5 array with 100 µm spacing between points. Each indentation was performed through 30 incremental loading steps, with a 0.2 s pause between increments, followed by a 2 s hold at the peak load of 50 mN, and then unloading in 30 steps. Hardness and elastic modulus values of the enamel surface were determined using NanoSuite software (Agilent, Santa Clara, CA, USA), which generated real-time force-displacement curves based on the load-displacement relationship. The Oliver-Pharr method was applied to derive hardness and elastic modulus values for each point, and Origin 2023 software (Origin Lab, Northampton, MA, USA) was used to generate distribution maps of these values across the enamel surfaces.

### Statistical analysis

All data were analyzed using the Statistics software (version 22; IBM SPSS Statistics, NY, USA, version 22). Data distribution was assessed using a Kolmogorov–Smirnov test. Statistical significance of the difference between groups was determined using Student’s t-test with a significance level of α<0.05.

## Results

### Clinical and radiographic features of case 1

**Figure 1:**
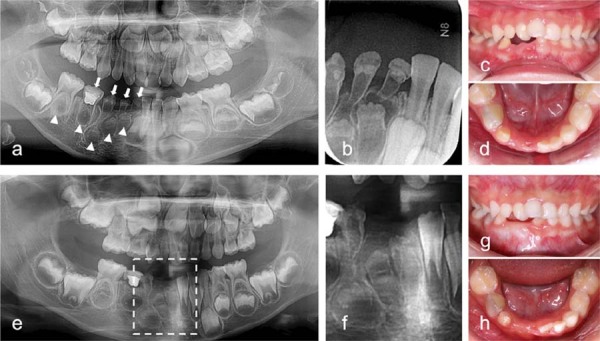
Radiographic and clinical presentation of a 4-year-old girl with ROD in the mandibular right quadrant. (a, b) Initial assessment: Panoramic (a) and periapical (b) radiographs taken on the first visit. White arrows highlight the affected primary teeth, whereas arrowheads indicate potentially involved permanent teeth. (c, d) Post-extraction intraoral views: Clinical images showing the mandibular right quadrant following the extraction of teeth 81 and 82 due to severe mobility. (e, f) Two-year follow-up imaging: Panoramic radiograph (e) and a magnified localized view (f) showing the developmental progress and radiographic changes in the region. (g, h) Two-year follow-up intraoral views: Clinical images indicating the soft tissue and dental status of the affected region at the two-year mark.

A 4-year-old girl presented to our hospital complaining of tooth mobility and pain. Her parents reported delayed eruption and recurrent gingival swelling of her mandibular right anterior teeth over the past year. The patient exhibited good health and had no family history of dental anomalies. Her mother did mention using contraceptives in the early stages of pregnancy, although the specific medication was not recalled. Intraoral examination showed that the mandibular primary right anterior teeth (teeth 81–83) had a yellowish color, small crown sizes, and rough surfaces. Teeth 81 and 82 exhibited grade III mobility and a gingival fistula. Panoramic and periapical radiographs revealed thin, hypocalcified layers of enamel and dentin, wide pulp chambers, and short roots in teeth 81–84 (Figure 1a-b). Tooth 84 was previously restored after undergoing pulpotomy. Delayed development of the permanent successors (teeth 41–45) and decreased bone mineral density of the right mandible were noted compared to the contralateral side. Additionally, analysis noted a supernumerary tooth in the maxillary anterior region, and the maxillary right permanent lateral incisor was missing. Teeth 81 and 82 were diagnosed with ROD and periapical periodontitis, and subsequently extracted due to pain and severe mobility (Figure 1c-d). The remaining ROD-affected primary teeth were preserved for long-term monitoring with periodic fluoride application. Teeth 83 and 84 showed no symptoms after two years (Figure 1g-h). Radiographic examination revealed root resorption in tooth 84 (Figure 1e), whereas the crown and root dentin of tooth 83 had thickened (Figure 1f), and its apex was more closed. The affected permanent teeth continued to develop but very slowly.

### Clinical and radiographic features of case 2

**Figure 2:**
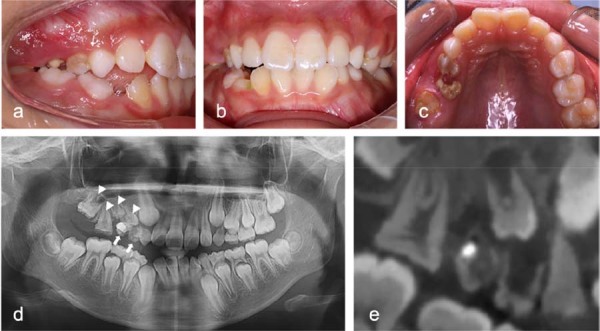
Radiographic and clinical presentation of a 9-year-old girl with ROD in the maxillary right quadrant. (a-c) Intraoral findings: (a) Buccal view showing severe caries and gingival inflammation associated with teeth 54 and 55. (b) Frontal occlusal view showing the overall dental status. (c) Palatal view highlighting the hypoplastic and yellowish discoloration of tooth 16, a characteristic ROD feature. (d, e) Radiographic evaluation: (d) Panoramic radiograph and (e) CBCT scan revealing root resorption and periapical radiolucency in primary teeth 54 and 55 (indicated by arrows). Permanent teeth 14 to 17 exhibit reduced crown size, thinner enamel and dentin layers, lower mineral density, and delayed developmental stages compared with their contralateral counterparts.

A 9-year-old girl was referred to our hospital complaining of delayed eruption of the upper right permanent first molar and gingival pain in the maxillary right quadrant. Her parents disclosed no family history of dental abnormalities. Upon clinical examination, teeth 54 and 55 showed severe decay, grade three mobility, and mild gingival swelling (Figure 2a-c). Tooth 16 was partially erupted, whereas tooth 55 had incomplete restorations. Teeth 54, 55, and 16 presented a yellowish color and rough surface texture. Panoramic radiography and cone-beam computed tomography imaging detected root resorption and periapical radiolucency in teeth 54 and 55 (Figure 2d-e). Additionally, the permanent posterior teeth (specifically, teeth 14–17) exhibited reduced crown dimensions, decreased mineral density, thinner enamel and dentin layers, enlarged pulp chambers, and delayed developmental stages compared to the contralateral counterparts. The patient was diagnosed with ROD and periapical periodontitis. The proposed treatment plan involved extracting the affected primary teeth and monitoring the eruption of the permanent teeth.

### Macroscopic features of the affected teeth

**Figure 3:**
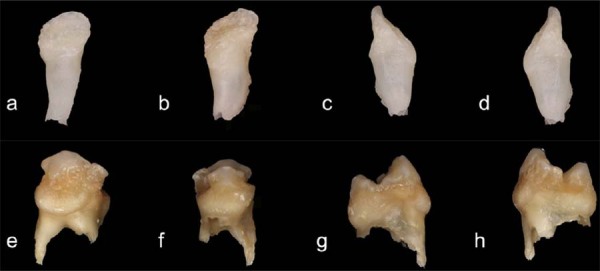
Macroscopic examination of extracted ROD-affected primary teeth. (a-d) Tooth 82 from Case 1: Sequential views of the labial, lingual, mesial, and distal surfaces, respectively. The tooth exhibits an irregular surface texture, hypoplastic enamel, and abnormal morphology. (e-h) Tooth 54 from Case 2: Corresponding sequential views showing severe structural defects, hypomineralized enamel, and an overall dysplastic appearance, characteristic of ROD-affected primary molars.

Figure 3 presents macroscopic images of the affected teeth, specifically tooth 82 from Case 1 and tooth 54 from Case 2. Tooth 82 presented yellowish discoloration, irregular morphology, and an open apex. Conversely, tooth 54 exhibited brownish discoloration, a rough surface texture, carious lesions, and pronounced root resorption.

### High-resolution micro-CT

**Figure 4:**
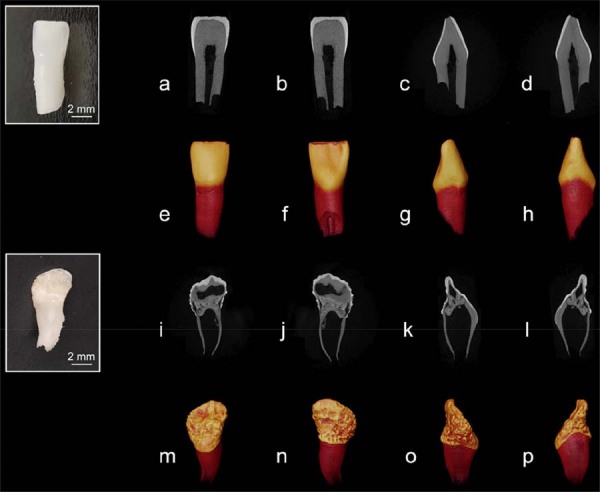
Micro-CT analysis of a control and a ROD-affected primary incisor. (a-h) Sagittal plane micro-CT images (a-d) and 3D reconstructed images (e-h) of a healthy primary incisor. (i-p) Sagittal plane micro-CT images (i-l) and 3D reconstructed images (m-p) of the ROD-affected primary incisor.

Figure 4 presents the high-resolution micro-CT images and the reconstructed 3D images of the control and ROD-affected primary incisors. The control tooth showed a smooth, flat crown surface characterized by physiological wear at the incisal edge (Figure 4a-h). Enamel thickness was relatively uniform, ranging from approximately 0.11 to 0.65 mm, and gradually thinned as it approached the cementoenamel junction. The enamel exhibited high radiodensity and a distinct demarcation from the dentin layer. Dentin layer thickness ranged from 1.00 to 2.70 mm, with the incisal area being the thickest. Root dentin maintained a consistent thickness, averaging approximately 1.10 mm, and presented partial resorption. Conversely, the ROD-affected tooth exhibited an irregular, shell-like morphology characterized by numerous pits and grooves on its surface (Figure 4i-p). The enamel layer was notably thin and uneven, measuring approximately 0.34 mm at the incisal edge and reducing to about 0.06 mm at the lingual cervical region, with certain areas of the crown surface showing reduced radiodensity.

The ROD-affected dentin presented porous structures with extensive voids, cracks, and fissures. Coronal sections exhibited internal cavities and fissures, some of which extended to the pulp chamber. The dentin structure in the lingual cervical region was similar to the control, displaying a uniform thickness of about 0.38–0.65 mm. Porous structures connecting to the normal dentin exhibited radiodensity akin to that of normal dentin. Root dentin presented a significantly reduced thickness, averaging approximately 0.25 mm. This was associated with an enlarged pulp chamber, widened apical foramina, and scattered calcifications. Quantitative analysis confirmed significantly reduced mineral densities in both enamel and dentin of ROD-affected teeth versus controls (*P*<0.05) (Table 1).

**Table 1:** Mineral density, enamel lattice parameters in enamel, and enamel element analysis of control and ROD-affected primary incisors (means±SD)

		Control	ROD
Mineral density (g/cm^3^)	Enamel	1.65±0.08	1.40±0.17*
	Dentin	0.92±0.02	0.82±0.03*
Lattice parameters in enamel (Å)	a	9.35	9.42
	c	6.88	6.88
Enamel element analysis (weight ratio)	Ca/P	1.900±0.100	1.770±0.200*
	Mg/Ca	0.007±0.004	0.013±0.009*

* Significant differences between ROD and control tooth (*P*<0.05)

### XRD analysis

**Figure 5:**
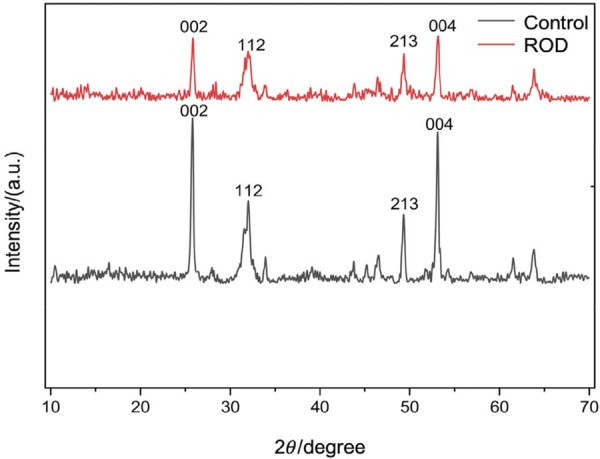
XRD patterns of enamel from control and ROD-affected primary incisors.

Figure 5 presents the enamel XRD patterns of ROD-affected and control primary incisors. The spectra of both specimens exhibited typical peak positions of hydroxyapatite. Peaks of the (002), (112), (213), and (004) planes in the ROD enamel were blunter and weaker than those in the control, indicating reduced crystallinity in ROD enamel. Lattice parameter *a* for ROD-affected crystallites was higher than that for the control, whereas parameter *c* remained consistent for both groups (6.88 Å), as calculated by the equation of hexagonal crystalline structures (Table 1). Significant lower crystallinity was evident in the ROD group (71.4%) compared to control (84.5%) according to the analysis using MDI Jade 6 software (MDI, Livermore, USA).

### SEM and EDS analysis

**Figure 6:**
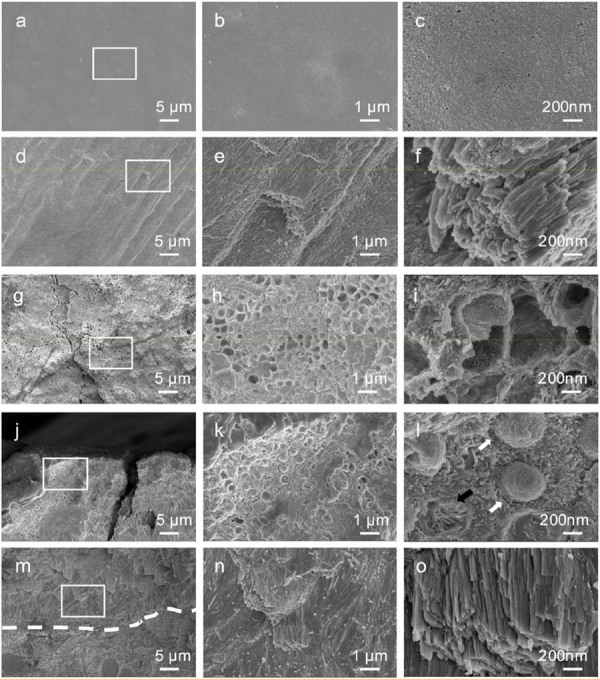
SEM images of enamel from control and ROD-affected primary incisors. (a-c) Enamel surface morphology of the control primary incisor: (a) Low-magnification SEM image showing the overall surface structure. (b, c) Higher magnifications of the boxed area in (a) revealing a smooth, compact, and well-mineralized enamel surface. (d-f) Fractured enamel cross-section of the control primary incisor: (d) Low-magnification SEM image showing the internal enamel structure. (e, f) Higher magnifications of the boxed area in (d) revealing organized enamel prisms and a dense, well-mineralized structure. (g-i) Enamel surface morphology of the ROD-affected primary incisor: (g) Low-magnification SEM image revealing a hypocalcified, poorly organized enamel structure with a honeycomb-like appearance. (h, i) Higher magnifications of the boxed area in (g) showing irregularly oriented enamel prisms and porous enamel with disorganized mineralization. (j-l) Outer enamel fractured surface of the ROD-affected primary incisor: (j) Low-magnification SEM image showing structural defects in the outer enamel layer. (k, l) Higher magnifications of the boxed area in (j) revealing hemispherical calcified structures (white arrows) and irregularly oriented enamel prisms (black arrow), indicative of abnormal mineralization and prism disorganization. (m-o) Inner enamel layer near the dentinoenamel junction (DEJ) of the ROD-affected primary incisor: (m) Low-magnification SEM image showing a transition between the outer and inner enamel layers, with the DEJ marked by a dashed line. (n, o) Higher magnifications of the boxed area in (m) revealing a more organized prism arrangement compared to the outer enamel but still displaying signs of mineralization abnormalities. Magnifications: (a, d, g, j, m) 2,000×; (b, e, h, k, n) 10,000×; (c, f, i, l, o) 50,000×.

SEM images of the control enamel revealed a smooth surface with densely packed, rod-shaped crystals (Figure 6). The enamel rods were arranged in parallel on the fractured surface, and the crystals within each rod appeared to be tightly and orderly aligned (Figure 6d–f). Conversely, the enamel surface of the ROD-affected primary tooth showed a rough, hypoplastic texture, exhibiting honeycomb-like structures (Figure 6g-h) with irregularly oriented enamel prisms (Figure 6i). The fractured surface of ROD-affected enamel revealed hypoplastic crystals within the outer enamel layer appearing as hemispheric or irregularly oriented prisms (Figure 6j–l). In contrast, the enamel layer proximal to the dentinoenamel junction displayed prismatic crystals organized more uniformly (Figure 6m–o).

**Figure 7:**
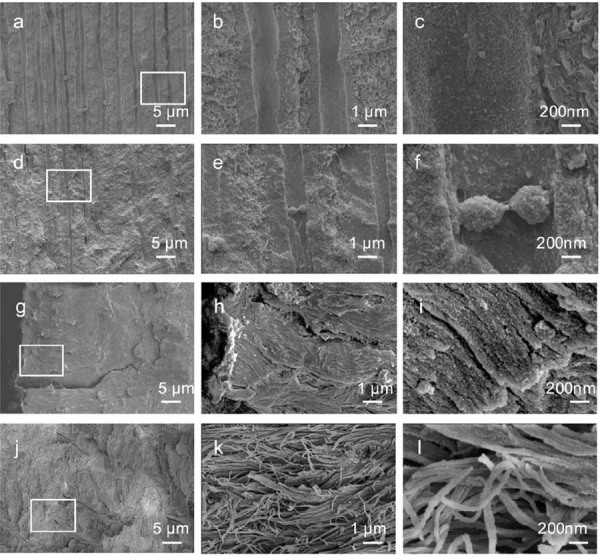
SEM images of dentin and cementum from control and ROD-affected primary incisors. (a-c) Control dentin: (a) Low-magnification SEM image showing a well-organized structure with clearly defined dentinal tubules. (b, c) Higher magnifications of the boxed area in (a) revealing the tubular architecture and homogeneous mineralization pattern characteristic of healthy dentin. (d-f) ROD-affected dentin: (d) Low-magnification SEM image displaying a disorganized dentin structure with thinner and sparser distribution of dentin tubules. (e, f) Higher magnifications of the boxed area in (d) showing spherical, and poorly mineralized hydroxyapatite crystals in the peritubular and intertubular dentin. (g-i) Control cementum: (g) Low-magnification SEM image showing well-organized cementum structure. (h, i) Higher magnifications of the boxed area in (g) revealing densely packed and perpendicularly oriented Sharpey’s fibers, indicative of a normal attachment apparatus. (j-l) ROD-affected cementum: (j) Low-magnification SEM image showing structural abnormalities in the cementum. (k, l) Higher magnifications of the boxed area in (j) revealing twisted, disorganized, and sparsely arranged Sharpey’s fibers, suggesting impaired structural integrity and attachment function. Magnifications: (a, d, g, j) 2,000×; (b, e, h, k) 10,000×; (c, f, i, l) 50,000×.

SEM images of the control dentin revealed a well-defined tubular structure within the intertubular matrix, characterized by uniformly oriented dentin tubules with consistent density and dimensions (Figure 7a). The intertubular matrix exhibited a rough, collagen-rich area, whereas the mineral-rich peritubular dentin appeared smoother at higher magnifications (Figure 7c-d). In contrast, the distribution of dentin tubules in the ROD-affected tooth was not uniform, appearing thinner and sparser (Figure 7d). The peritubular dentin was rough and poorly mineralized hydroxyapatite crystals were found in the peritubular and intertubular dentin (Figure 7e–f).

A laminated structure was observed in the cementum of the control tooth, characterized by the presence of numerous Sharpey’s fibers penetrating the cementum perpendicular to the root surface (Figure 7g–i). Although the ROD-affected cementum maintained a comparable appearance to the control, its fiber arrangement was twisted and less dense (Figure 7j–l).

According to the elemental analysis results (Table 1), the Ca/P ratio of enamel in the affected tooth was significantly lower than that of the control (*P*<0.05), while the Mg/Ca ratio presented the opposite trend (*P*<0.05).

### TEM analysis

**Figure 8:**
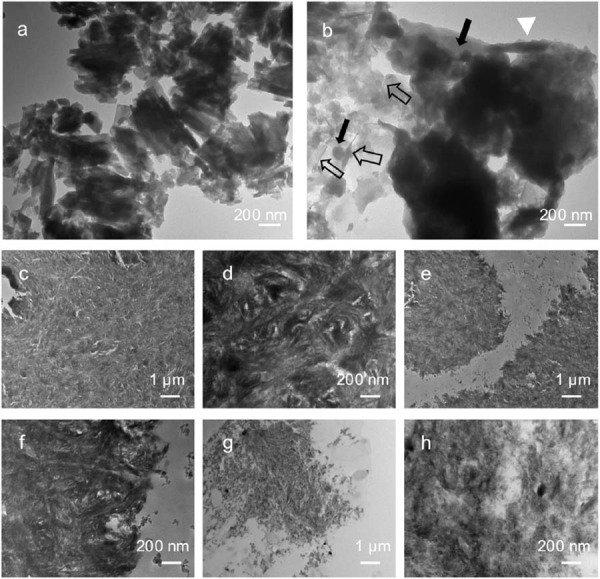
TEM images of enamel and dentin from control and ROD-affected primary molars. (a) TEM image of control enamel showing well-arranged, long, lath-like hydroxyapatite crystals. (b) TEM image of ROD-affected enamel revealing a disorganized mineralization pattern with a heterogeneous mix of crystal morphologies: rod-like (white triangle), needle-like (hollow arrows), and round-shaped (black arrows). (c) TEM image of control dentin showing a highly organized and well-mineralized collagen matrix with fibrils intricately interwoven in bundles. (d) Boxed area in (c) at higher magnification highlights the dense and uniform mineralization of collagen fibrils. (e-h) ROD-affected dentin displays two distinct structural zones. (e-f) Well-mineralized zone: (e) TEM image showing a relatively organized collagen structure with mineral deposition. (f) Boxed area in (e) at higher magnification reveals irregular mineralization within collagen fibrils. (g-h) Hypocalcified zone: (g) TEM image showing a poorly mineralized region with disorganized fibrils. (h) Boxed area in (g) at higher magnification demonstrates loosely arranged and sparsely mineralized collagen fibrils, indicative of impaired dentin mineralization. Magnifications: (a, b, c, e, g) 10,000×; (d, f, h) 50,000×.

TEM images revealed that the enamel of the control primary molar consisted of well-packed long, lath-like crystals with about 30~80 nm in width (Figure 8a). In contrast, the enamel of the ROD-affected primary molar showed poorly crystallized, randomly oriented and aggregated crystals which displayed three distinct morphologies: rod-like, needle-like, and round-shaped (Figure 8b). Control dentin presented uniformly dense dentinal tubules characterized by intricately interwoven bundles of highly mineralized collagen fibrils. Needle-shaped hydroxyapatite crystals were embedded within these collagen fibrils, maintaining their well-organized structure (Figure 8c-d). Conversely, ROD-affected dentin displayed two distinct structural characteristics: one comprised bundles of densely mineralized collagen fibrils (Figure 8e-f), while the other showed loosely organized, needle-shaped hydroxyapatite crystals (Figure 8g-h).

### Nanoindentation analysis

**Figure 9:**
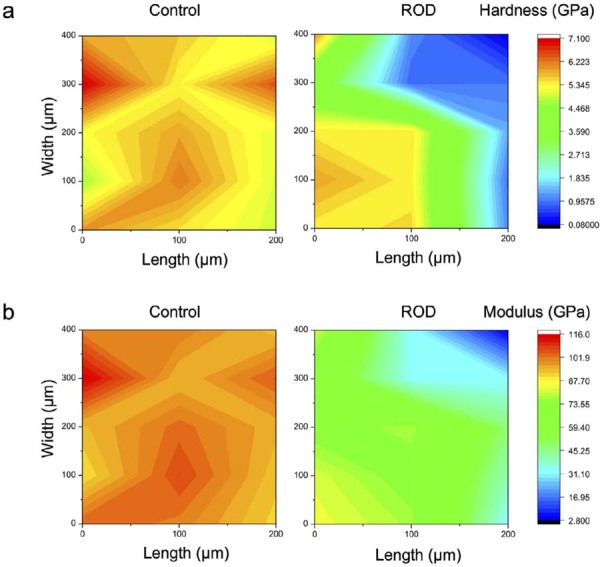
Hardness and elastic modulus distribution maps of enamel from control and ROD-affected primary incisors. (a) Hardness distribution. (b) Elastic modulus distribution.

Figure 9 presents the nanoindentation results of the enamel surfaces of ROD-affected and control primary incisors. The enamel in the control primary incisor showed relatively uniform hardness and elastic modulus, with hardness values ranging from 4.96 to 7.09 GPa and an elastic modulus ranging from 89.9 to 115.6 GPa. Conversely, specific areas of the ROD-affected incisor enamel surface (marked in blue-green) presented reduced hardness, with values between 0.093 and 1.30 GPa, and a correspondingly low elastic modulus, ranging from 2.90 to 47.97 GPa. Some affected enamel regions, indicated in orange-yellow, exhibited hardness values between 3.70 to 6.03 GPa and an elastic modulus between 66.47 and 84.49 GPa, comparable to the properties observed in control.

## Discussion

ROD is a rare developmental anomaly with hallmarks of hypoplasia and hypocalcification of dental hard tissues which causes defects that usually impact both the crown and root structures of primary and permanent teeth. Its exact etiology remains unidentified. This study examined two ROD cases, neither of which had a family history of dental abnormalities. Contraceptive use by the mother in the early stages of pregnancy emerged as a potential contributing factor in the first case, whereas disease etiology in the second case remained unknown. These observations highlight the need for further research to investigate the interplay between genetic and environmental factors that may contribute to ROD development. ROD generally presents in a localized manner, often affecting one or two jaw quadrants, common in the maxilla rather than the mandible. The reported cases were confined to a single jaw quadrant (right maxilla and right mandible), affecting both primary and permanent teeth within those quadrants. These localized traits align with previous findings suggesting that ROD may be more prevalent in certain dental arch regions, potentially associated with localized developmental disturbances during odontogenesis.

High-resolution micro-CT analysis revealed that the enamel and dentin of the affected teeth presented extensive mineralization defects and a significantly reduced mineral content (Figure 4), supporting previous findings.^14,15^ Our results regarding the irregular crown surface and the presence of thin, hypocalcified enamel align with the observations by Lustmann and Ulmansky^16^ (1976) and Gardner^19^ (1974). Concerns are caused by the porous nature of the dentin, along with internal cracks and grooves extending from the dentin to the pulp, as these features may facilitate bacterial invasion thereby increasing the risk of pulpitis and periapical periodontitis.^19,20^

According to Bartlett^21^ (2013), enamel formation primarily involves the secretory and maturation phase. The secretory phase is dominated by ameloblast-mediated secretion of enamel matrix which determines enamel thickness and structural morphology, whereas the maturation phase is responsible for mineralizing and crystallizing the secreted enamel matrix. Thus, the enamel defects observed in ROD-affected teeth, including uneven enamel thickness, irregular morphology, and hypomineralization, may indicate disruptions during both the secretory and maturation phase of enamel development. These developmental abnormalities are consistent with the uneven enamel observed in ROD-affected teeth and imply that ameloblast dysfunction plays a key role in the ROD pathogenesis. A hypothesis further supported by the XRD data (Figure 5) and SEM analyses (Figure 6g-o), which revealed alterations in enamel crystallinity and mineral composition. These changes may be linked to the substitution of ions such as CO_3_^2^ and Mg^2^ within the hydroxyapatite crystals.^22,23^ These structural changes, including reduced crystallinity and altered crystal lattice parameters, may contribute to the mechanical instability of the enamel, rendering it more prone to wear and fracture.

Control dentin presented well-defined peritubular and intertubular structures, with consistently oriented, dense tubules and highly mineralized collagen fibrils embedded with organized hydroxyapatite crystals (Figure 7g-i). Conversely, ROD-affected dentin showed thinner and sparser dentin tubules, and the hydroxyapatite crystals in the peritubular and intertubular dentin were poorly mineralized (Figure 7j-l). These findings align with previous reports.^15,19,20,24^ TEM further elucidated two distinct patterns in ROD-affected dentin: densely packed collagen bundles (Figure 8e-f) and loosely arranged, disorganized hydroxyapatite crystals (Figure 8g-h). These results suggest that although the developmental disturbances in ROD are extensive, they may not equally affect every region of the tooth. This implies that ROD-affected teeth may retain some developmental potential, particularly if they are preserved at an early developmental stage. Moreover, the cementum of ROD-affected teeth exhibited certain similarities to that of control teeth but also some structural irregularities, such as sparser and twisted Sharpey’s fibers (Figure 7j-l). These abnormalities may be linked to chronic periapical inflammation and disruptions in normal tooth eruption processes.^14,25^

EDS analysis revealed significant alterations in elemental composition, particularly an increased Mg/Ca ratio and a decreased Ca/P ratio in ROD-affected enamel (Table 1). Elevated Mg^2^⁺ levels are known to inhibit Ca^2^⁺ during biomineralization, thereby affecting hydroxyapatite crystal formation.^26^ These compositional changes may contribute to the increased susceptibility to dental caries observed in clinical cases.^27^

Nanoindentation testing showed substantial reductions in the hardness and elastic modulus of ROD-affected enamel compared with control (Figure 9). These diminished mechanical properties correspond with the observed structural abnormalities, suggesting that ROD-affected enamel is more prone to fracture and wear. The reduced enamel hardness and elasticity, coupled with the presence of porous dentin, may account for the increased susceptibility to caries and other dental complications.^28^ These findings highlight the importance of implementing preventive treatment strategies for individuals with ROD like fluoride therapy and routine monitoring to reduce the risk of dental caries and maintain the function of affected teeth.

Management of ROD-affected teeth remains debatable, particularly regarding the timing and necessity of extractions. Some clinicians advocate for the early extraction of ROD-affected teeth due to their developmental defects and heightened risk of infection.^29^ Early extraction may mitigate complications associated with pulpitis and periapical periodontitis and decrease the frequency of necessary dental interventions. Conversely, other researchers warn that premature extractions may lead to long-term adverse outcomes such as facial asymmetry and insufficient bone development for future dental implants.^30,31^ In this study, all affected teeth were preserved except those with uncontrolled periapical inflammation, severe root resorption, or grade III mobility. After a two-year follow-up, notably, the mandibular right primary canine in case 1 exhibited increased hard tissue thickness, radiodensity, and apical closure, suggesting that ROD-affected teeth may retain some developmental potential with proper preservation and management. These findings suggest that with appropriate care and monitoring, certain ROD-affected teeth may retain adequate function and development to fulfill their role within the dental arch.

## Conclusion

Within the limitations of the present study, ROD-affected primary teeth display notable structural, mechanical, and chemical abnormalities. The identified defects in enamel, dentin, and cementum highlight the necessity for targeted treatment strategies that address the particular challenges presented by ROD-affected teeth. Early intervention, preventive care, and regular monitoring are essential for managing ROD effectively and preserving dental function. Further investigation into the genetic and environmental factors contributing to the disease, alongside the development of novel therapies to promote mineralization and restore mechanical properties, is imperative for improving long-term outcomes in ROD-affected individuals.

## References

[1] Hamdan MA, Sawair FA, Rajab LD, Hamdan AM, Al-Omari IKH (2004). Regional odontodysplasia: a review of the literature and report of a case. Int J Paediatr Dent.

[2] Tervonen SA, Stratmann U, Mokrys K, Reichart PA (2004). Regional odontodysplasia: a review of the literature and report of four cases. Clin Oral Investig.

[3] Alotaibi O, Alotaibi G, Alfawaz N (2019). Regional odontodysplasia: an analysis of 161 cases from 1953 to 2017. Saudi Dent J.

[4] Magalhães AC, Pessan JP, Cunha RF, Delbem AC (2007). Regional odontodysplasia: case report. J Appl Oral Sci.

[5] Nijakowski K, Wos P, Surdacka A (2022). Regional odontodysplasia: a systematic review of case reports. Int J Environ Res Public Health.

[6] Rushton MA (1965). Odontodysplasia: "ghost teeth". Br Dent J.

[7] Chaudhry AP, Wittich HC, Stickel FR, Holland MR (1961). Odontogenesis imperfecta. Report of a case. Oral Surg Oral Med Oral Pathol.

[8] Koskinen S, Keski-Filppula R, Alapulli H, Nieminen P, Anttonen V (2019). Familial oligodontia and regional odontodysplasia associated with a PAX9 initiation codon mutation. Clin Oral Investig.

[9] Zegarelli EV, Kutscher AH, Applebaum E, Archard HO (1963). Odontodysplasia. Oral Surg Oral Med Oral Pathol.

[10] Burch MS, Besley KW, Samuels HS (1973). Regional odontodysplasia with associated midline mandibular cyst: report of case. J Oral Surg.

[11] Herold RC, Lally ET, Gold L (1976). Abnormal tooth tissue in human odontodysplasia. Oral Surg Oral Med Oral Pathol.

[12] Pinkham JR, Burkes EJ (1973). Odontodysplasia. Oral Surg Oral Med Oral Pathol.

[13] Walton JL, Witkop CJ, Walker PO (1978). Odontodysplasia. Report of three cases with vascular nevi overlying the adjacent skin of the face. Oral Surg Oral Med Oral Pathol.

[14] Carlos R, Contreras-Vidaurre E, Almeida OP, Silva KR, Abrahão PG, Miranda AMMA (2008). Regional odontodysplasia: morphological, ultrastructural, and immunohistochemical features of the affected teeth, connective tissue, and odontogenic remnants. J Dent Child.

[15] Kerebel B, Kerebel LM (1982). Structural, ultrastructural, microradiographic, and electron-probe studies of an unusual case of regional odontodysplasia. J Dent Res.

[16] Lustmann J, Ulmansky M (1976). Structural changes in odontodysplasia. Oral Surg Oral Med Oral Pathol.

[17] Rohanizadeh R, Pouëzat J, Bohne W, Ajacques JC (1998). Ultrastructural organization and microanalysis studies of deciduous enamel crystallites in regional odontodysplasia (RO). J Oral Pathol Med.

[18] Shahmoradi M, Swain MV (2016). Quantitative characterization and micro-CT mineral mapping of natural fissural enamel lesions. J Dent.

[19] Gardner DG (1974). The dentinal changes in regional odontodysplasia. Oral Surg Oral Med Oral Pathol.

[20] Ide M, Oshima Y, Chiba T, Adaniya A, Kuroki T, Miake Y (2023). Histological findings of regional odontodysplasia in maxillary right region in two cases. Pediatr Dent J.

[21] Bartlett JD (2013). Dental enamel development: proteinases and their enamel matrix substrates. ISRN Dent.

[22] Bazin D, Chappard C, Combes C, Carpentier X, Rouzière S, André G (2009). Diffraction techniques and vibrational spectroscopy opportunities to characterise bones. Osteoporos Int.

[23] LeGeros RZ, Sakae T, Bautista C, Retino M, LeGeros JP (1996). Magnesium and carbonate in enamel and synthetic apatites. Adv Dent Res.

[24] Luder HU (2015). Malformations of the tooth root in humans. Front Physiol.

[25] Müller WE, Neufurth M, Ushijima H, Muñoz-Espí R, Müller LK, Wang S (2022). Molecular and biochemical approach for understanding the transition of amorphous to crystalline calcium phosphate deposits in human teeth. Dent Mater.

[26] Aoba T, Moreno EC, Shimoda S (1992). Competitive adsorption of magnesium and calcium ions onto synthetic and biological apatites. Calcif Tissue Int.

[27] Robinson C, Weatherell JA, Hallsworth AS (1981). Distribution of magnesium in mature human enamel. Caries Res.

[28] Angker L, Nockolds C, Swain MV, Kilpatrick N (2004). Correlating the mechanical properties to the mineral content of carious dentine-a comparative study using an ultra-micro indentation system (UMIS) and SEM-BSE signals. Arch Oral Biol.

[29] Hess P, Lauridsen EF, Daugaard-Jensen J, Worsaae N, Kofod T, Hermann NV (2020). Treatment strategies for patients with regional odontodysplasia: a presentation of seven new cases and a review of the literature. Oral Health Prev Dent.

[30] Barbería E, Sanz Coarasa A, Hernández A, Cardoso-Silva C (2012). Regional odontodysplasia. A literature review and three case reports. Eur J Paediatr Dent.

[31] Cunha JL, Santana AV, Santana LA, Santos DM, Amorim KS, Maciel Souza LA (2020). Regional odontodysplasia affecting the maxilla. Head Neck Pathol.

